# Effect of dehydrofluorination reaction on structure and properties of PVDF electrospun fibers†

**DOI:** 10.1039/d1ra05667k

**Published:** 2021-09-15

**Authors:** Yuxin Wang, Haijun Wang, Kun Liu, Tong Wang, Chunlei Yuan, Haibo Yang

**Affiliations:** Shaanxi University of Science and Technology Xi'an 710021 Shaanxi China wanghaijun@sust.edu.cn

## Abstract

Piezoelectric nanosensors were prepared with a novel type of dehydrofluorinated poly(vinylidene fluoride) (PVDF) nanofibrous membrane. With the synergistic effect of the dehydrofluorination reaction and applied high voltage electric field, the piezoelectric and energy storage properties of fibrous membranes attained great improvement. It was found that the simultaneous introduction of conjugated double bonds to the backbone of PVDF which was accompanied with the elimination of HF, resulted in the decrease of its molecular weight, solution viscosity and hydrophobicity. The crystalline phase, diameter, piezoelectric and energy storage properties of electro-spun PVDF nanofiber membranes significantly depend on the degree of HF elimination in dehydrofluorinated PVDF. The dehydrofluorinated PVDF with 5 hours of reaction exhibits the highest discharged energy density (*W*_rec_) and energy storage efficiency (*η*), but excessive dehydrofluorination reaction is unfavorable to the energy storage properties. In addition, the dehydrofluorinated PVDF fiber membrane-based nanosensor possesses a larger electrical throughput (open circuit voltage of 30 V, which is three time that of the untreated PVDF), indicating that the introduction of double bonds can also improve the piezoelectric properties of PVDF nanofibers.

## Abstract 2

A piezoelectric nanosensor was prepared with a novel type of dehydrofluorinated poly(vinylidene fluoride) (PVDF) nanofibrous membrane.

## Graphical abstract

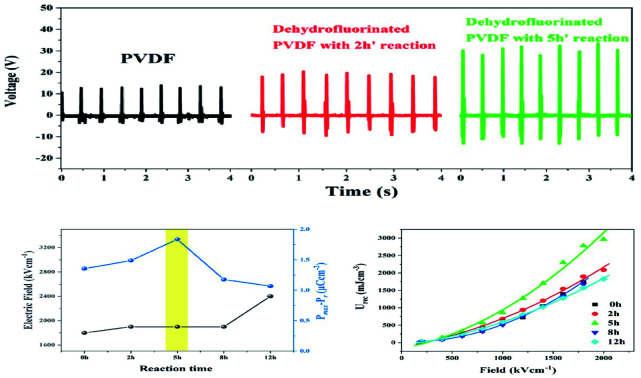

## 1. Introduction

Piezoelectric sensors have attracted widespread attention in recent years and have been widely applied in energy harvesting,^1–3^ human health monitoring,^4^ physiological activities,^5^ and medical diagnosis.^6,7^ The structure of a piezoelectric sensor is mainly composed of a flexible substrate, active materials, and conductive electrodes, among which active piezoelectric materials are the most important part with excellent mechanical flexibility, piezoelectricity, conductivity, sensitivity, and high energy storage.^8,9^ Piezoelectric materials mainly include inorganic ceramics and organic polymers. Although inorganic ceramics like barium titanate (BaTiO_3_) possess excellent electromechanical conversion and strong spontaneous polarization, their inherent brittleness makes them unsuitable for direct integration into flexible devices.^10,11^ On the other hand, the piezoelectric polymer represented by poly(vinylidene fluoride) (PVDF) and its copolymers, can be used as an ideal raw material for preparing flexible piezoelectric sensors owing to their good flexibility and piezoelectric properties.^12^

PVDF can crystallize into at least three different types of crystalline phases.^13,14^ Generally speaking, PVDF crystallizes from the melt and then forms an electrically inactive α-phase with a conformation of TGTG′ which cannot be polarized. The crystalline phase with TTTGTTTG′ conformation is described as the γ-phase whose molecular chain includes a certain amount of G conformation, greatly reducing the polarity of γ-phase. Compared with the α- and γ-phase PVDF, the β-PVDF with all-*trans* TTT molecular chain conformation shows the highest polarization performance and the most favourable ferroelectric and piezoelectric properties. Therefore, how to produce high content β-phase and improve its properties has always been a matter of great concern.^15–18^

It is well known that copolymerization is an efficient way to produce β-PVDF with TTT conformation. The introduction of comonomers with a large steric hindrance such as trifluoroethylene (TrFE), chloride trifluoride ethylene (CTFE), hexafluoropropene (HFP) and others increases the conformational potential energy of the TGTG′ conformation chains in the α-phase structure.^19–21^ As a result, β-phase with TTT conformation is always favored regardless of processing method. To further suppress the energy loss of β-phase under zero electric field, some rigid polymer chains such as polystyrene (PS),^22^ polymethacrylate (PXMA)^23^ and polyacrylonitrile^24^ have also been attached to PVDF-based polymer chains. Through these chemical modifications, the normal ferroelectric behavior of β-PVDF can be turned into relaxation or ferroelectric character with the narrowing of displacement–electric field (*D*–*E*) loops, therefore increasing the energy density and breakdown strength of PVDF membranes. However, the complicated copolymerization process, largely decreased Curie temperature and elastic modulus of PVDF membranes remain a concern.

Different from the copolymerization or grafting reaction with the addition of comonomers, the dehydrofluorination reaction adopts the subtraction strategy to adjust the molecular conformation. In this way, cheap ethylenediamine or other organic bases was used to remove HF from PVDF and carbon–carbon double bonds were simultaneously introduced to the main chains of PVDF molecules. As is reported by Sodano *et al.*, the introduced C=C double bond in dehydrofluorination reaction also possesses a strong steric hindrance effect on the TGTG′ conformation chains while having negligible influence on the all-*trans* TTT conformation chains, thereby increasing the content of β-phase and inhibiting the crystallization of α-phase. As a result, the thermal stability, energy-conversion efficiency and piezoelectric properties of PVDF can be greatly improved.^25^

The orientation of all *trans* β-PVDF crystals is another key factor affecting the piezoelectric properties. Studies have found that the oriented β-phase crystals with TTT conformation can be produced by applying external fields, such as mechanical stretching and electrospinning, among which electrospinning is a promising technology.^26–28^ During the electrospinning process, PVDF molecular chains are forced to stretch along the direction of electric field force and adopt the TTT conformation, which contributes to the crystallization and orientation of β-phase nanocrystals. Meanwhile, the electric dipole –CH_2_/–CF_2_ orients perpendicularly to the fiber axis in high voltage electrostatic field, which allows β-phase PVDF nanofibers to be used directly to build piezoelectric sensors without the need of post-polarization. Deep investigation has been conducted into the electrospinning of PVDF-TrFE or other PVDF copolymers.^21^ Compared with PVDF homopolymer, electrospun PVDF-TrFE nanofibers present more excellent piezoelectric properties. However, to our knowledge, no study has reported the structure and property of dehydrofluorinated PVDF nanofibers. Taken into consideration the synergistic effects of chemical modification with organic bases and applied electric field, it is desirable that the energy-conversion efficiency and piezoelectric performance of dehydrofluorinated PVDF nanofibers will show significant improvement.

In this paper, the appearance, crystalline structure and properties of dehydrofluorinated PVDF fibrous membranes have been reported in detail. The molecular structure of dehydrofluorinated PVDF and the corresponding viscosity of PVDF solution were measured by X-ray photoelectron spectroscopy (XPS) and rheometer, respectively. The effect of dehydrofluorination reaction on the morphology and crystalline structure of PVDF fibers was investigated by scanning electron microscopy (SEM), X-ray diffraction (XRD) and Fourier transform infrared (FTIR) spectroscopy. For investigation of the energy storage property of the dehydrofluorinated PVDF fibrous membranes, hysteresis loops were applied to calculate the energy storage efficiency and density. Finally, the dehydrofluorinated PVDF fibrous membranes were prepared into a piezoelectric sensor with a constant force study on its piezoelectric performance.

## 2. Experimental section

### 2.1. Materials and sample preparation

The PVDF used in the experiments, with its weight-average molecular mass of 250 000 g mol^−1^, was purchased from Sigma-Aldrich Reagent Co. Ltd., China.

The dehydrofluorinated PVDF was prepared as previously reported.^25^ The PVDF pellets (20 g) was dissolved in DMF (100 ml) and stirred at 60 °C for 3 h to obtain a homogeneous PVDF/DMF solution. As the dehydrofluorination agent, the ethylenediamine powder (5 g) was added into the PVDF/DMF solution, and then stirred at 30 °C. The reaction solution was extracted at different time (*e.g.*, 2, 5, 8, 10, 12 and 48 h) and was then coagulated in deionized water. The obtained DHF-PVDF films were dried in a vacuum oven at 25 °C for 12 h to ensure the removal of residual ethylenediamine and DMF.

The 20% w/v spinning solutions were prepared through dissolving of dehydrofluorinated PVDF films in a mixed solvent of DMF and acetone (4 : 6 by v/v), and was stirred at 50 °C for 3 h. Then the solutions were kept at room temperature for 24 h to sufficiently eliminate bubbles. Pour 4 ml solution into a 10 ml hypertonic syringe with a diameter of 0.8 mm. A positive high voltage of 20 kV was employed between the needle and collector, with the feed rate of the syringe pump of 1.0 ml h^−1^. The collector was positioned 15 cm away from the needle, and aluminum foil was utilized as a collector. The process continued for 4 h. Finally, the obtained nanofibers were dried at 25 °C for 12 h to ensure the solvents to be released completely.

The pressure sensor, with a size of 1.0 cm × 2.0 cm, was fabricated by sandwiching the dehydrofluorinated fibrous membranes between two copper electrodes. Then, copper wires were attached onto the top and the bottom of the electrodes by silver paste. In the end, the polyamide films were used for encapsulation outside the film and electrode.

### 2.2. Characterization

The chemical structure of the untreated and DHF-PVDF was analyzed by X-ray photoelectron spectroscopy (XPS, AXIS SUPRA, with an Al K alpha X-ray source, UK) and UV-Vis spectroscopy (PerkinElmer, Lambda 750) in the range of 200–800 nm. The morphologies of dehydrofluorinated PVDF nanofibers were investigated by scanning electron microscopy (SEM, MAGELLAN-400). FTIR spectra were recorded at a resolution of 2 cm^−1^ and 32 scans from 1600 to 400 cm^−1^ were averaged. The XRD data were obtained by X-ray diffraction (XRD, D8 Advance) at a scanning rate of 1° min^−1^ from 10° to 30° with a step interval of 0.02°. Contact angle is measured by video optical contact angle measuring instrument (OCA20). The stress–strain curves were recorded by testing machine (Instron-5940) at a stretching rate of 5 mm min^−1^. The pressure response performance of the fabricated sensor was measured by a digital oscilloscope (TBS 1152B). A Radiant Premier II Ferroelectric Test System was used to test the electric polarization–electric field (*P*–*E*) hysteresis loops at a frequency of 10 Hz.

## 3. Results and discussion

### 3.1. Dehydrofluorination reaction

As is reported by Sodano *et al.*, through the well-controlled dehydrofluorination (DHF) reaction with ethylenediamine, hydrogen fluoride (HF) can be eliminated from PVDF and carbon–carbon double bonds are simultaneously introduced to the PVDF backbone. As an efficient method, XPS can provide the fraction of double bonds to quantify the extent of the reaction. Therefore, XPS was also applied to track the progress of the dehydrofluorination reaction of PVDF. Considering the important factors that affect the subsequent electrospinning process, we further studied the molecular weight and distribution of dehydrofluorinated PVDF with different reaction times, as well as the viscosity of the corresponding solutions.

**Fig. 1:**
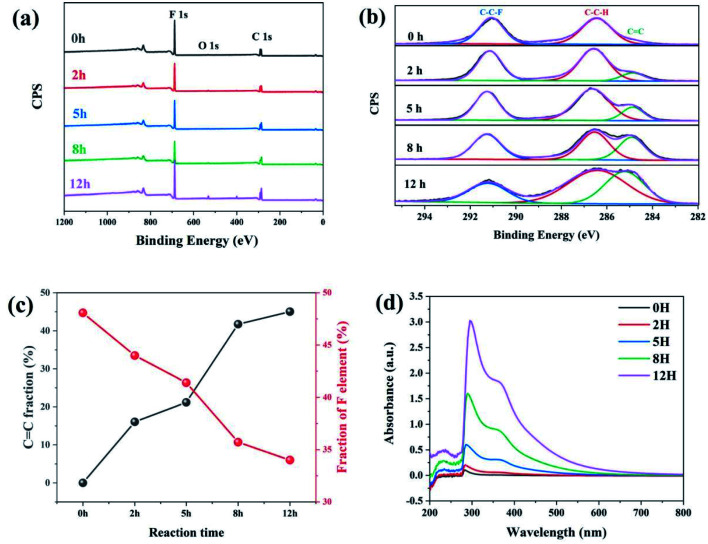
(a) The survey XPS spectra, (b) the C 1s spectra, (c) C=C and F element fraction, and (d) UV transmittance spectra of dehydrofluorinated PVDF with varying reaction times.

Fig. 1a shows the survey XPS spectra of dehydrofluorinated PVDF within 12 h. The F 1s and C 1s peaks appeared at 684 and 282 eV, respectively.^29^ The fractions of fluoride and carbon atoms in different samples can be calculated according to the integral of these peak areas. As is seen in Fig. 1c, with the prolongation of reaction time, the content of F element decreased from 48.1% for untreated PVDF to 34.0% for 12 h, which confirms that fluoride was successfully eliminated from PVDF by its reaction with ethylenediamine.

Fig. 1b shows the XPS C 1s spectra of dehydrofluorinated PVDF with different reaction times. As can be seen from Fig. 1b, the characteristic energy level peak at 284.6 eV corresponding to the C=C double bond became stronger with the extension of reaction time.^29^ The fraction of C=C double bonds varying with reaction times was calculated and shown in Fig. 1c. The obtained results shows that the fraction of C=C double bonds increased monotonously from 0% for pristine PVDF to 45.2% for 12 h. What's more, with the elimination of F element, C=C double bonds were gradually introduced into the molecular chains of PVDF, which was consistent with the report of Sodano, *et al.*^25^

Whether these newly introduced double bonds are conjugated or not may have a great influence on the conformation adjustment of PVDF molecular chains and the properties of produced fibers in the electrospinning process. Consequently, the UV-visible absorption spectra of dehydrofluorinated PVDF with different reaction times were then measured to find out whether these double bonds were conjugated. Fig. 1d shows the UV spectra of dehydrofluorinated PVDF with different reaction times within the wavelength range of visible light. As shown in Fig. 1d, the fiber membranes with reaction time of 0 h, 2 h, 5 h, 8 h and 12 h absorbed light in the UV and visible regions, with absorption maxima of 371, 367, 362, 359 and 355 nm, respectively. Compared with untreated PVDF fiber membranes, their redshift amplitude was 4–16 nm. It indicates that the introduction of carbon–carbon double bond through dehydrofluorinated reaction makes the modified PVDF have conjugated structure.^30^ In other words, as the dehydrofluorination reaction proceeded, more rigid conjugated double bonds were introduced into the molecular chains of PVDF.

The number-average (*M*_n_), weight-average (*M*_w_) molecular weight and its distribution (*M*_w_/*M*_n_) of dehydrofluorinated PVDF were used to represent the breakage or crosslinking of molecular chains. Fig. S1† shows the GPC chromatogram of dehydrofluorinated PVDF with different reaction times. As is seen in Fig. S1,† all dehydrofluorinated PVDF samples with different reaction times revealed a single peak with elution time, which was slightly prolonged with the increase of reaction time. The calculated *M*_w_, *M*_n_ and *M*_w_/*M*_n_ were summarized in Table 1. It was found that *M*_w_ decreased from 250 000 to 183 000 g mol^−1^ when the reaction time increased from 0 h to 12 h. Furthermore, the molecular weight distribution (*M*_w_/*M*_n_) did not increase significantly with reaction times (see Table 1), which indicates that the decrease in molecular weight should be mainly ascribed to the elimination of HF instead of the breakage of molecular chains.^31^

**Table 1:** Molecular weight of the dehydrofluorinated PVDF with varying reaction times

Sample	Reaction time (h)	*M*_n_ (g mol^−1^)	*M*_w_ (g mol^−1^)	*M*_w_/*M*_n_
1	0	139 000	250 000	1.79
2	2	131 000	248 000	1.89
3	5	123 000	212 000	1.71
4	8	117 000	198 000	1.68
5	12	107 000	183 000	1.71

**Fig. 2:**
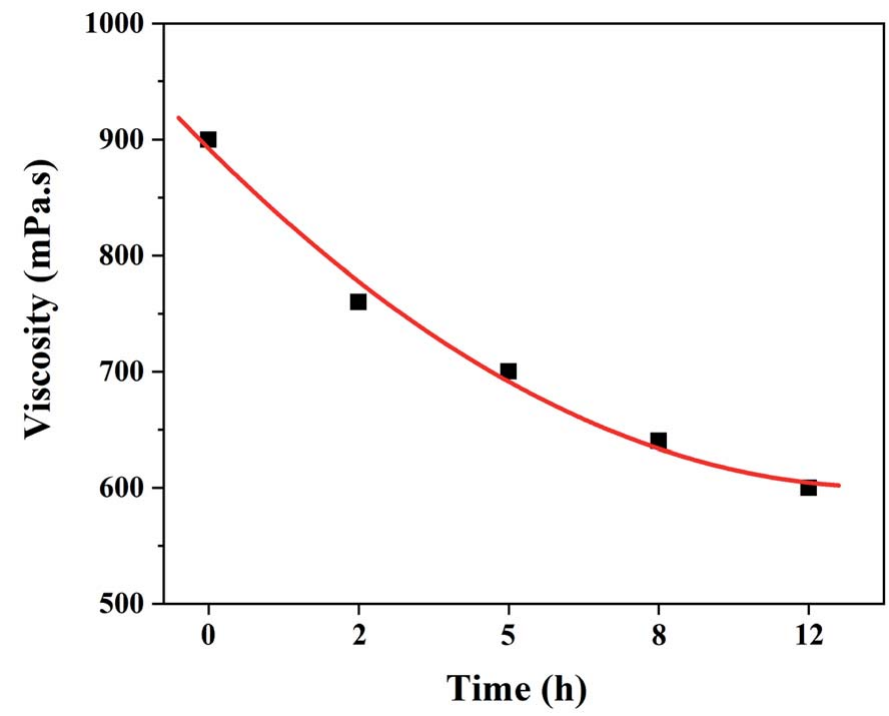
Solution viscosity of the dehydrofluorinated PVDF with varying reaction times.

It is well known that the viscosity of electrospinning solution is not only related to the stability of electrospinning process, but also has an important influence on the morphologies and structure of the resultant fibers.^32^ Therefore, it is necessary to investigate the effect of dehydrofluorinated reaction on the viscosity of PVDF solutions. Fig. 2 shows the viscosity of the dehydrofluorinated PVDF solution varying with the reaction time. It was found that the viscosity of PVDF solution showed a monotonous decrease with the extension of reaction time. For instance, the viscosity of PVDF solution was about 900 mPa s before the dehydrofluorination reaction, but it decreased to 600 mPa s after 12 h. As is confirmed by Fig. 1, more and more rigid conjugated double bonds were introduced to PVDF chains with the reaction time, which may weaken the chain entanglement between PVDF molecules and then decrease the solution viscosity.^31^ It can be obtained in the experimental observation that the dissolution rate of dehydrofluorinated PVDF within 10 h was significantly faster than that of pristine PVDF. It suggests that the dehydrofluorinated reaction weakened the interaction between the molecular chains, thus improving the solubility of PVDF.

### 3.2. Morphology and crystalline structure of dehydrofluorinated PVDF nanofibers

**Fig. 3:**
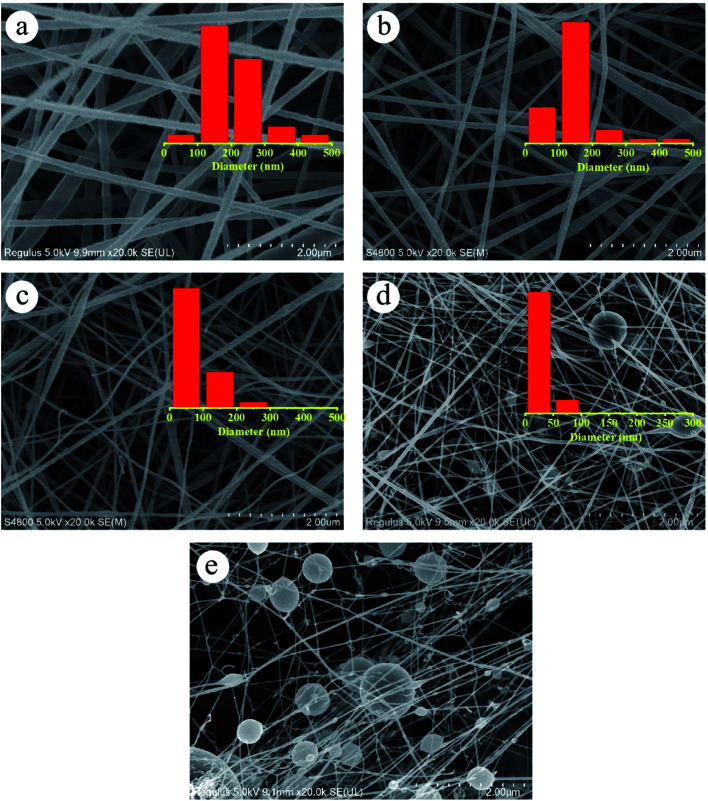
SEM images of the nanofibers spun with PVDF dehydrofluorinated for (a) 0 h, (b) 5 h, (c) 8 h, (d) 10 h and (e) 12 h.

The effect of dehydrofluorinated reaction on the structure and properties of electrospun PVDF nanofibers was then investigated. Fig. 3 shows SEM images of PVDF nanofibers electrospun from the dehydrofluorinated solution of PVDF with different reaction time. It can be clearly seen that the diameter of dehydrofluorinated PVDF nanofiber gradually decreased with the increase of reaction time. The average fiber diameter of pristine PVDF was 190 ± 10 nm (inset of Fig. 3a), while the average diameter of dehydrofluorinated PVDF nanofibers with 10 h reaction decreased to 40 ± 10 nm (inset of Fig. 3d). Accordingly, the content of element F decreased from the initial 48.1% to 35.2%, which was shown in Fig. 1c. As is shown in Fig. 3d and e, when the reaction time was beyond 10 h, some of the nanofibers were broken and many spherical beads were simultaneously produced. This demonstrates that the fiber diameter, morphology, and mean pore size strongly depend on the degree of eliminating HF in dehydrofluorinated PVDF used in the electrospinning process. It is suggested that the formation of broken fibers and beads is ascribed to the decreased chain entanglement.^32^ As was discussed in Section 3.1, the elimination of HF and the introduction of double bonds weakened the intermolecular interaction and chain entanglement in solution, which was characterized by the reduced viscosity. Once the weakened entanglement force was not strong enough to stabilize the electrified jet and thus to suppress the Rayleigh instability, the molecular chains would slip under the drawing force in the electrostatic field, finally resulting in broken fibers and beads.

**Fig. 4:**
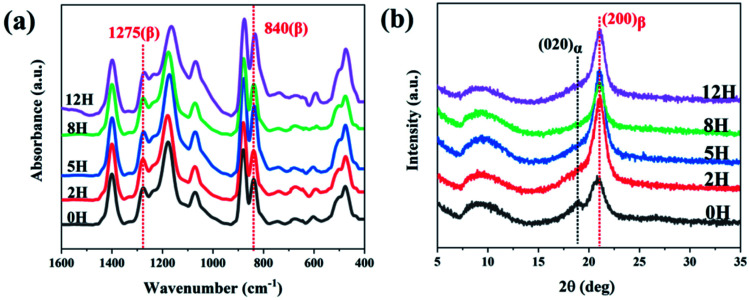
(a) FTIR spectra and (b) XRD profiles of dehydrofluorinated PVDF nanofibers with varying reaction times.

FT-IR spectra and WAXD patterns were used to show the synergic effect of dehydrofluorinated reaction and electrostatic field on the crystalline phases of PVDF nanofibers. As it is reported, the characteristic FT-IR absorptions of α-PVDF appear at 610, 766, 796 and 976 cm^−1^, and those of β-PVDF appear at 510, 840 and 1275 cm^−1^.^13^ As could be seen in Fig. 4a, dehydrofluorinated PVDF nanofibers with varying reaction times presented the vibration bands of β-PVDF and those of α-PVDF disappeared. In the process of electrospinning, PVDF molecular chain was drawn along the electric field direction under the electric field force and finally formed TTT conformation, which was beneficial to the crystallization and orientation of β-phase crystals but inhibited the generation of α-phase.^25^ In addition, the dehydrofluorinated reaction can further increase the content of β-phase. As is shown in Table S1,† the fraction of β-phase increased from 83.3% to 92.1% as the reaction time increased from 0 h to 5 h. However, excessive double bonds will exert side effect. As is also seen in Table S1,† the content of β-phase decreased to 59.9% in dehydrofluorinated PVDF nanofibers with 24 h reaction. This is in good agreement with the study of Sodano *et al.* They found that the conformational potential energy in the β-phase decreases while that in the α-phase increases after a certain fraction of carbon–carbon double bonds is introduced to the PVDF backbone. However, with the extension of reaction time, the introduction of too many molecular defects undoubtedly weakens the crystallization ability of PVDF, thereby reducing the content of β-phase.

The XRD results further confirmed the induction effect of eliminating HF on β-PVDF. As is shown in Fig. 4b, the untreated PVDF showed a strong diffraction peak at 21.6° and a weak one at 18.4°. The former corresponded to the (200) reflection of β-phase while the latter corresponded to the (020) reflection of α-phase, which suggests that a small number of α-phase crystals appear in the untreated PVDF nanofibers.^14^ However, dehydrofluorinated PVDF fibers showed only the diffraction peak of β-phase, indicating that the dehydrofluorinated modification greatly promote the crystallization of β-phase but inhibits the crystallization of α-phase.

In conclusion, the application of untreated PVDF in electrospinning enabled the drafting effect of electric field force to promote the generation of β-PVDF to a certain extent, but there still existed a small amount of α-phase crystals. On the contrary, when dehydrofluorinated PVDF was used for electrospinning, the double bonds introduced into the PVDF molecular chain and the drafting and orientation of electric field force formed a synergistic effect, jointly promoting the generation of β-PVDF, and making the content of β-phase in the prepared fiber membrane increase to 92.1%.

### 3.3. Physical properties

#### 3.3.1. Wettability behavior

**Fig. 5:**
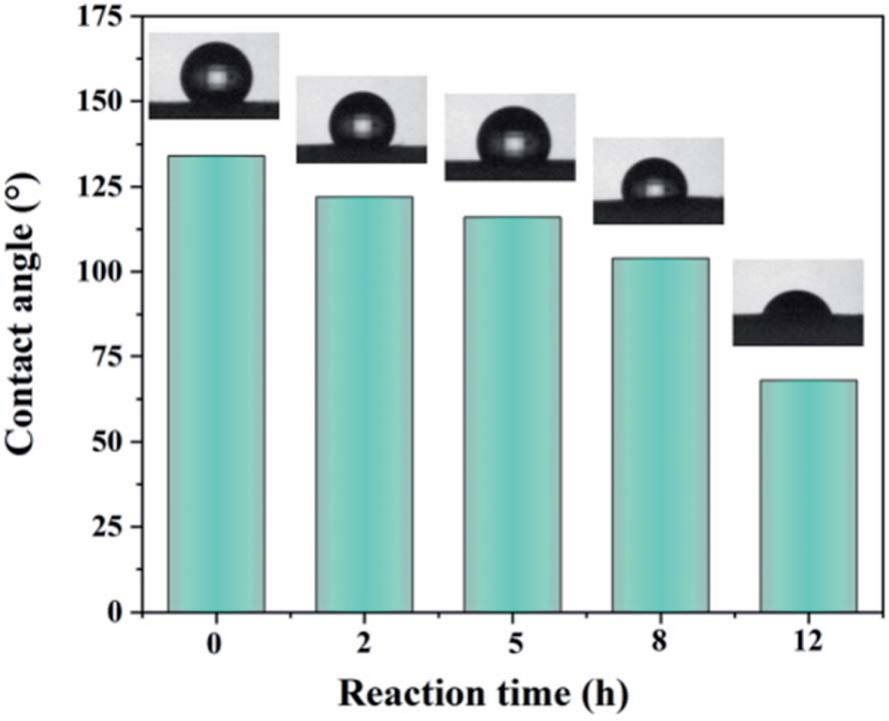
Contact angle of dehydrofluorinated PVDF fiber membrane with varying reaction times.

Typically, the decrease of fiber diameter in a membrane can reduce the volume of air pockets and increase their number per unit area, thus increasing the contact angle. However, as is seen from Fig. 5, the contact angle of water on the surface of dehydrofluorinated PVDF fiber membranes decreased from the initial 135° to 75° with the reaction time increasing from 0 h to 12 h, indicating that dehydrofluorinated modification increased the surface energy of PVDF. In order to eliminate the influence of pores between fibers on surface energy, the solution casting films of dehydrofluorinated PVDF with different reaction times were then prepared. DI water, ethylene glycol, and glycerol were used as the testing liquids. According to the Young equation and the Lewis acid/base theory,^33–35^ the surface energy of dehydrofluorinated PVDF with different reaction times was calculated and shown in Table 2 (the detailed calculation method is shown in ESI†). As can be seen from Table 2, the surface energy of PVDF increased from 22.4 mJ m^−2^ to 38.6 mJ m^−2^ as the reaction time increased from 0 h to 12 h.

**Table 2:** Surface energy of dehydrofluorinated PVDF films with varying reaction times

Reaction time	0 h	2 h	5 h	8 h	12 h
Surface energy (mJ m^−2^)	22.4	25.2	27.5	31.8	38.6

#### 3.3.2. Mechanical properties

**Fig. 6:**
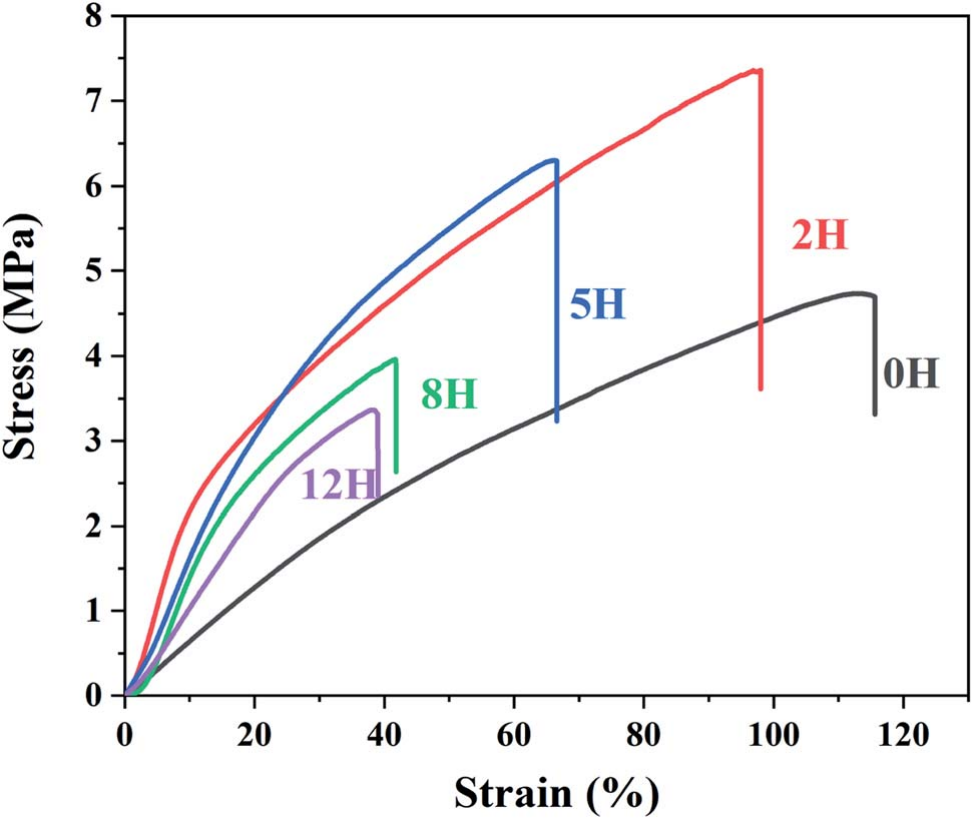
Stress–strain curves of dehydrofluorinated PVDF fiber membrane with varying reaction times.

Fig. 6 shows the strain–stress curves of the pristine and dehydrofluorinated PVDF fibrous membranes. As is seen from Fig. 6, the strength at break increased from 4.5 MPa for the pristine PVDF to 7.2 MPa for the dehydrofluorinated PVDF with 2 hours' reaction, and decreased with longer reaction time. For the PVDF that have reacted within 5 h, the introduction of a small number of double bonds may be conducive to the orientation of the molecular chains in the electric field, thus improving the tensile strength of PVDF fibrous membranes. In addition, the moderate decrease of fiber diameter may also enhance the physical cross-linking points between the fibers, which also helps to increase the tensile strength of the membranes.^36^ With the extension of reaction time, more broken fibers and beads are formed, which weakens the physical cross-linking between the fibers and leads to the decrease in the tensile strength of the membranes. As is also seen from Fig. 6, the elastic modulus of dehydrofluorinated PVDF fiber membranes was obviously higher than that of pristine PVDF. Additionally, the elongation at break decreased from 116.5% for the pristine PVDF to 98.4% for the dehydrofluorinated PVDF with 2 hours' reaction, and further decreased with the extension of reaction time. The elongation is only about 39.6% when the reaction time is 12 hours. It indicates that the gradually weakened chain entanglement and increased conjugated C=C double bonds reduces the toughness and increases the elastic modulus of the dehydrofluorinated PVDF fibrous membranes.^37^

#### 3.3.3. Energy storage performance

**Fig. 7:**
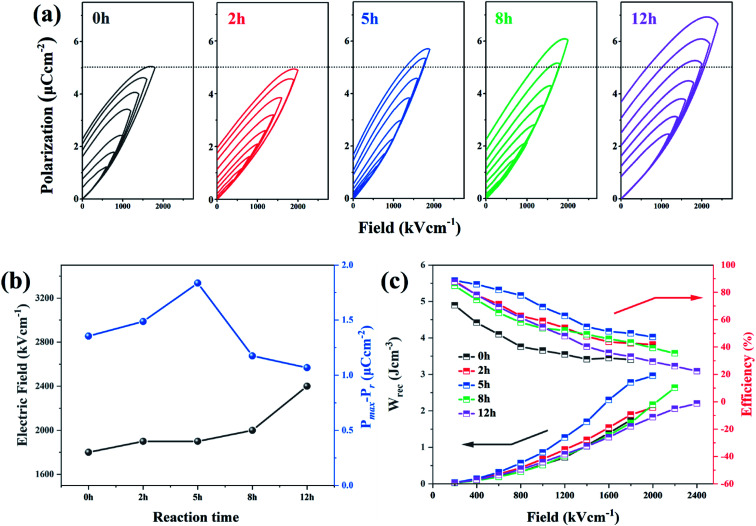
(a) Hysteresis loops, (b) breakdown strength and *P*_max_ − *P*_r_ values (c) discharged energy density (*W*_rec_) and efficiency (*η*) of dehydrofluorinated PVDF fiber membranes with varying reaction times.

Fig. 7a represents the typical polarization–electric field (*P*–*E*) hysteresis loops (measured at 100 Hz with various electric fields) of the dehydrofluorinated PVDF with 0 h, 2 h, 5 h, 8 h and 12 h, respectively. From Fig. 7a, it was found that the breakdown strength of dehydrofluorinated PVDF fibers increased monotonically from 1800 kV cm^−1^ to 2400 kV cm^−1^ with the reaction time increasing from 0 h to 12 h. However, the polarization of the dehydrofluorinated PVDF fiber membrane with varying reaction times, which can be described by maximum electric displacement minus remanent electric displacement (*P*_max_ − *P*_r_), increased first and then decreased with reaction time prolonging. For example, at an electric field of 1000 kV cm^−1^, *P*_max_ − *P*_r_ increased from 1.355 to 1.836 μC cm^−2^ with the reaction time increasing from 0 h to 5 h, and then decreased with reaction time prolonging (Fig. 7b). The enhanced electric displacement should be attributed to the increasing dipole polarization and improved breakdown strength of dehydrofluorinated PVDF nanofibers.^38^

The enhancement of electric displacement of the nanofiber further gives rise to the increasing discharged energy density, which is usually described as *W* = ∫*E*d*D*, where *E* is the electric field and *D* is the electric displacement.^39,40^ The energy storage density (*W*_rec_) and energy storage efficiency (*η*) of the pristine PVDF and its dehydrofluorinated counterpart were then calculated by integrating the different parts of the *P*–*E* loop and the result is shown in Fig. 7c. From Fig. 7c, it was found that both *W*_rec_ and *η* of the dehydrofluorinated PVDF increased at first and then decreased as reaction time prolonged. At all electric field, the dehydrofluorinated PVDF with 5 hours' reaction exhibits the highest *W*_rec_ (2.96 J cm^−3^ at an electric field of 1800 kV cm^−1^) and *η* (49.29% at an electric field of 1800 kV cm^−1^). This is consistent with the changing trend of *P*_max_ − *P*_r_.

#### 3.3.4. Piezoelectric properties

**Fig. 8:**
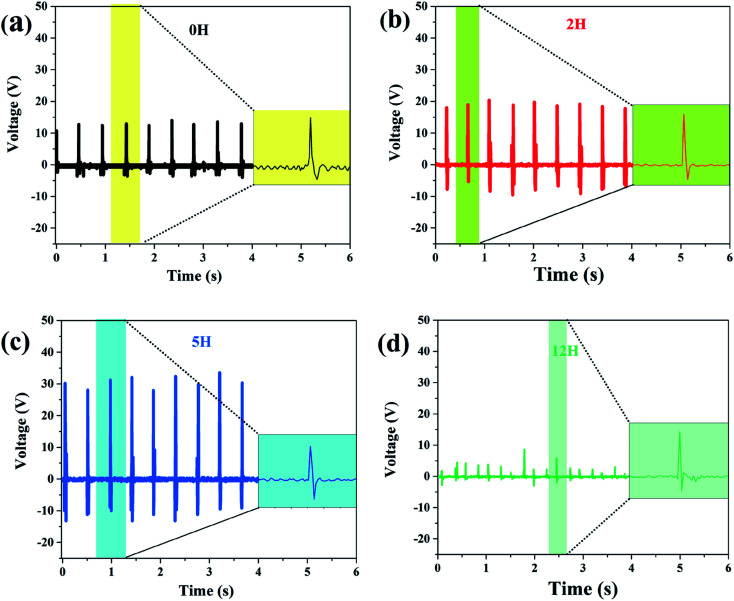
Measured output voltage of the sensor made of dehydrofluorinated PVDF under continuous finger bending. The reaction time of dehydrofluorinated PVDF is (a) 0 h, (b) 2 h (c) 5 h and (d) 12 h.

The fibrous membrane of dehydrofluorinated PVDF with varying reaction times was made into sensors, and the corresponding piezoelectric response was studied in detail. Fig. 8 shows the output voltage of the sensors under continuous finger bending. As is seen from this figure, the output voltage increased from 12 V for pristine PVDF to 20 V for the dehydrofluorinated PVDF with 2 hours' reaction, and then increased to 30 V for the sample with 5 hours' reaction. The output voltage per unit area of the sensors was further calculated. It was shown that the output voltage per unit area increased from 6 V cm^−2^ for pristine PVDF to 10 V cm^−2^ for the dehydrofluorinated PVDF with 2 hours' reaction, and then increased to 15 V cm^−2^ for the sample with 5 hours. However, when the reaction time exceeded 5 h, the output voltage of the sensor decreased. Additionally, the sensitivity of the sensors made of dehydrofluorinated PVDF was also improved. As is seen from the inset figure in Fig. 8, the response time decreased from 0.012 s to 0.006 s when the reaction time increased from 0 h to 5 h. These results fully demonstrate that the dehydrofluorinated reaction can significantly improve the piezoelectric properties of PVDF nanofibers, potentially due to the increase in β-phase content and the decrease in fiber diameter. The dehydrofluorinated PVDF fibers with large aspect ratios and small diameters are easily deformed by external force.^41^ In addition, it was found that the fiber membranes which were produced with excessive dehydrofluorination PVDF exhibited poor piezoelectric response. For example, after 12 hours' reaction, it is difficult for the membrane to withstand continuous bending or knocking and is extremely easy to break, thus resulting in unstable output voltage of the sensor.

**Fig. 9:**
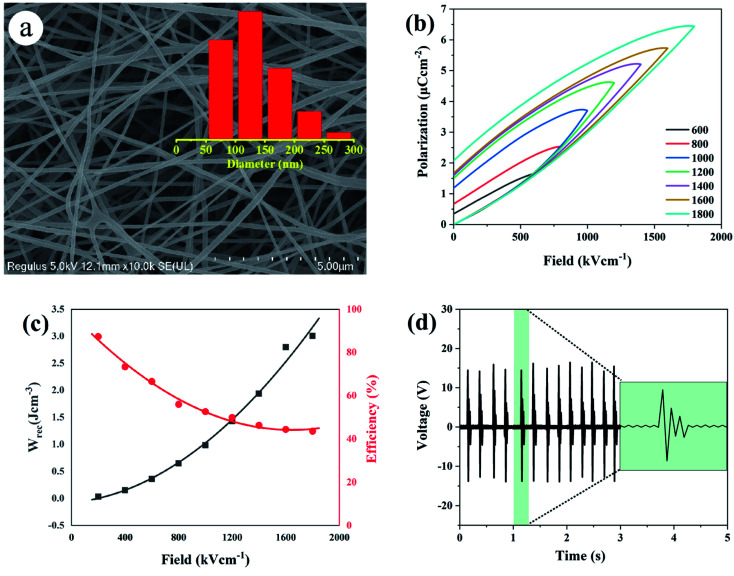
(a) SEM images, (b) *P*–*E* loops of dehydrofluorinated PVDF with varying time. (c) Stored energy density (*W*_rec_) and efficiency (*η*) of dehydrofluorinated PVDF with 12 h. (d) The output curve of the sensor made of dehydrofluorinated PVDF with 12 h.

One may doubt whether the decrease of ferro- and piezo-electric properties of PVDF fiber membranes is only caused by the decrease of solution viscosity, and further leads to the formation of many beads and broken fibers within the membrane. In order to clarify this problem, the solution concentration was increased from 20 to 25 wt% after 12 hours' reaction so as to improve the viscosity of the solution. Besides, the membrane without broken fibers and beads (see Fig. 9a) was obtained. As is expected, the mechanical, ferro- and piezo-electric properties of PVDF fiber membranes were well improved by increasing the solution viscosity. However, it should be noticed that both *U*_rec_ and *η* of the dehydrofluorinated PVDF with the reaction time of 12 h are still lower than those with the reaction time of 5 h (Fig. 9b and c). Additionally, the produced sensor can only generate a response voltage of 15 V, which is only half that of the sample reacting for 5 h (Fig. 9d). It clearly indicates that excessive reaction is unfavorable to the ferro- and piezo-electric properties because the crystallization ability of β-phase is weakened and the resultant crystalline structure becomes more disordered after the introduction of excessive double bonds, *i.e.*, molecular defects.

## 4. Conclusions

In this work, a novel type of dehydrofluorinated PVDF nanofibrous membrane was prepared through electrospinning method. The morphology, hydrophobicity, mechanical properties, piezoelectric and energy storage properties of the dehydrofluorinated PVDF nanofibrous membranes were studied. The results show that the properties of fibrous membranes are greatly improved with the synergistic effect of dehydrofluorinated reaction and applied high voltage electric field. Conjugated double bonds are simultaneously introduced to the backbone of PVDF accompanying with the elimination of HF, which results in the decrease of its molecular weight and solution viscosity. As a result, the diameter of dehydrofluorinated PVDF nanofibers decreases with the prolongation of reaction time. However, excessive dehydrofluorinated reaction leads to the formation of broken fibers and spherical beads, which is unfavorable to the formation of oriented β-PVDF crystals. Hence, the crystalline phase, piezoelectric and energy storage properties of electro-spun PVDF nanofiber membranes shows a trend of increase at first and then present a trend of decrease. The fibrous membrane of dehydrofluorinated PVDF with the reaction time of 5 h presents the best polarization, discharged energy density and energy efficiency. Additionally, a larger electrical throughput (open circuit voltage of 30 V, which is three times that of the untreated PVDF) from the dehydrofluorinated fiber membrane-based nanosensor indicates that the introduction of double bonds can also improve the piezoelectric properties of PVDF nanofibers. In consideration of the excellent piezoelectric and energy storage properties of the dehydrofluorinated PVDF fibrous membrane, the preliminary results explore an effective way to obtain a promising candidate material for the preparation of portable electronic devices.

## Conflicts of interest

The authors declare that they have no known competing financial interests or personal relationships that could have appeared to influence the work reported in this paper.

## Supplementary Material

RA-011-D1RA05667K-s001
