# Supplementary material for: Effect of dehydrofluorination reaction on structure and properties of PVDF electrospun fibers
Source: RSC Adv. 2021 Sep 15;11(49):30734–43. doi: 10.1039/d1ra05667k (PMC9041349; doi:10.1039/d1ra05667k)
Supplement: RA-011-D1RA05667K-s001 [file RA-011-D1RA05667K-s001.pdf]

Electronic Supplementary Material (ESI) for RSC Advances.
This journal is © The Royal Society of Chemistry 2021

Supporting Information for

Effect of Dehydrofluorinated Reaction on Structure and Properties of PVDF Electrospun Fibers

Yuxin Wang[1], Haijun Wang[1], Kun Liu[1], Chunlei Yuan[1], Tong Wang[1], Haibo Yang[1]

[1] Shaanxi University of Science and Technology, Xi'an 710021, Shaanxi, China

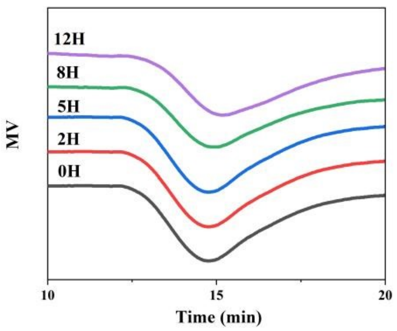

Figure S1. GPC chromatogram of dehydrofluorinated PVDF with varying reaction times.

Table S1. Fraction of β-phase in dehydrofluorinated PVDF nanofibers with varying reaction times.

**Time/h**	0	2	5	8	12	24
**F(β)**	83.3%	89.5%	92.1%	86.2%	84.6%	59.9%

Table S2. The total surface energy and their components of the testing liquid.

	γ^{LW} (mJ m^{-2})	γ^{+} (mJ m^{-2})	γ^{-} (mJ m^{-2})	γ_{L} (mJ m^{-2})
Water	21.8	25.5	25.5	72.8
Ethylene glycol	29.0	1.9	47.0	50.8
Glycerol	34.0	3.9	57.4	64.0

Table S3. Contact angle of dehydrofluorinated PVDF films with varying reaction times.

[1] Corresponding author. Shaanxi University of Science and Technology, Xi'an 710021, Shaanxi, China.

E-mail address: wanghaijun@sust.edu.cn

Contact angle (°) \ Reaction time	0 h	2 h	5 h	8 h	12 h
Water	102.2	98.5	91.7	87.6	78.8
Ethylene glycol	70.5	67.3	62.5	58.4	51.2
Glycerol	87.3	89.9	92.6	102.4	113.3

Supplementary Notes

The specific process of calculating surface energy and the theory are as follows:

(1) DI water, ethylene glycol, and glycerol were used as the testing liquids. The total surface energy and their components of three testing liquids are list in Tab. S2.[33,34]

(2) In the LW–AB model, the total surface energy is composed of a dispersion component γ^{LW} due to Lifshitz–vander Waals interactions (SE–LW) and an acid–base component γ^{AB} due to Lewis interactions (SE–AB). Therefore, the surface energy is expressed as:

$$\gamma = \gamma^{LW} + \gamma^{AB}$$

where the acid–base component γ^{AB} consists of two contributions, one from an electron acceptor γ^{+} and other from an electron donor γ^{-}. Accordingly, this component is written as:

$$\gamma^{AB} = 2\sqrt{\gamma^{+}\gamma^{-}}$$

(3) Furthermore, the liquid–solid interface energy is calculated as:

$$\gamma_{LS} = \gamma_{L} + \gamma_{S} - 2\left(\sqrt{\gamma_{L}^{LW}\gamma_{S}^{LW}} + \sqrt{\gamma_{L}^{+}\gamma_{S}^{-}} + \sqrt{\gamma_{S}^{+}\gamma_{L}^{-}}\right)$$

where γ_L and γ_S are the liquid and solid surface energy, respectively.

(4) Combining the Young equation, it is acquired that:

$$\left(\gamma_{L}^{LW} + 2\sqrt{\gamma_{L}^{+}\gamma_{L}^{-}}\right)(1+\cos\theta) = 2\left(\sqrt{\gamma_{L}^{LW}\gamma_{S}^{LW}} + \sqrt{\gamma_{L}^{+}\gamma_{S}^{-}} + \sqrt{\gamma_{S}^{+}\gamma_{L}^{-}}\right)$$

where θ is the contact angle of the films.
